# Nationwide Trends of Pediatric Obesity and BMI z-Score From 2017-2021 in China: Comparable Findings From Real-World Mobile- and Hospital-Based Data

**DOI:** 10.3389/fendo.2022.859245

**Published:** 2022-05-26

**Authors:** Yan Yang, Miao Zhang, Jian Yu, Zhou Pei, Chengjun Sun, Jingwei He, Tian Qian, Feihong Luo, Shaoyan Zhang, Zhenran Xu

**Affiliations:** ^1^ Department of Pediatric Endocrinology and Inherited Metabolic Diseases, National Children’s Medical Center, Children’s Hospital of Fudan University, Shanghai, China; ^2^ Shijiazhuang Xigao Technology Co. Ltd., Shijiazhuang City, China; ^3^ Department of Integrative Medicine, National Children’s Medical Center, Children’s Hospital of Fudan University, Shanghai, China; ^4^ Department of Clinical Nutrition, National Children’s Medical Center, Children’s Hospital of Fudan University, Shanghai, China

**Keywords:** pediatric obesity, body mass index, children, adolescent, China

## Abstract

**Introduction:**

Lifestyle changes including COVID-19 lockdown cause weight gain and may change obesity trends; however, timely changes are largely unknown and monitoring measures are usually lack. This first large-scale study aimed to analyze the real-world national trends of obesity prevalence of Chinese children in the past five years, and the impact of COVID-19 pandemic on pediatric obesity development through both mobile- and hospital-based data.

**Methods:**

This study included children aged 3 to 19 years old all over China from January 2017 to April 2021. Hospital-measured and parent-reported cases from XIGAO database were analyzed. Body mass index (BMI) z-score calculation and obesity status evaluation were made according to Chinese standards. We evaluated obesity/overweight prevalence over the past five years and the changes of BMI z-score during COVID-19 lockdown.

**Results:**

A total of 656396 children from 31 provinces were involved, including 447481 hospital-measured cases and 208915 parent-reported cases. The obesity and overweight prevalence were 8.05% (95%CI 7.76%–8.39%) and 10.06% (95%CI 10.79%–11.55%), comparable to those of China National Nutrition Surveys during 2015–2019. Northern China had the highest obesity prevalence. Parent-reported data had higher obesity/overweight prevalence than hospital-measured data (18.3% [95%CI 17.7%–18.9%] *vs.* 21.7% [95%CI 20.7%–23.0%]). The trend of obesity prevalence remained stable with slight decrease, but COVID-19 lockdown caused a significant increase of 1.86% in 2020. Both mobile- and hospital-based data showed weight gain in the first half of 2020. High BMI z-score increase were found among primary and junior middle school children, and children in northeast area during lockdown.

**Conclusion:**

Weight gain during COVID-19 among Chinese children had regional differences and mainly affect primary and junior middle school children, thus warrants targeted interventions. The mobile growth assessment based on parent-reported data was a feasible, efficient and timely way for obesity monitoring among Chinese children, especially during epidemic.

## Introduction

Obesity in children and adolescents is a challenging public health issue, affecting more than 120 million children and adolescents aged 5–19 years in 2016 (1). In the past 30 years, the national obesity prevalence of children aged 6–17 in China increased from 1.8%–2.4% in the 1990s to 7.9%–12.7% in 2015–2019 (2). Excessive calorie intake and inactivity and/or sedentary behavior increase the body mass index (BMI) and obesity prevalence of children, which not only lead to the risk of cardiovascular and metabolic diseases, but also cause psychological and social complications. The shutdown caused by COVID-19 pandemic restricted children and adolescents’ outdoor physical activities and changed their living habits including diet and sleep, resulting in weight gains (3). Both studies from China regional data (4) or other countries found significant weight gain during lockdown among school-aged children and adolescents (5).

Regular growth assessment is an important way to monitor weight gain of children. Monitoring data can not only help schools and governments to introduce corresponding countermeasures, but also remind parents in time to help their children develop a healthy lifestyle. With the popularization of mobile phones and the Internet, parents can use mobiles to upload data and assess the growth and development of their children anytime and anywhere. This contactless way has become a good method of monitoring during the epidemic without going to hospitals or schools.

Population-based study about weight changes of children across China before and after the COVID-19 pandemic was limited. This study aimed to evaluate the prevalence of obesity of children and adolescents of all age groups in various regions of China in the past five years; and the impact of the COVID-19 pandemic on obesity development among children of different regions and ages in the real-world. Furthermore, we aimed to evaluate the feasibility of mobile parental-evaluation in the growth and development monitoring of Chinese children.

## Methods

### Study Population

In order to evaluate the changes of growth of Chinese children and adolescents continuously in the real world, this study analyzed data from January 2017 to April 2021 from the XIGAO database, which was developed to monitor the characteristics and trend of Chinese children’s growth and development. XIGAO database was developed by Shijiazhuang Xigao Technology Co., Ltd, which consisted of two parts of data, part based on hospitals and the other based on mobiles. The hospital part of the database was built in 2016, and the data began to be collected from mobiles in 2017. Therefore, this study only included data after 2017. More than 1500 hospitals in 29 provinces, autonomous regions or municipalities were involved in the database. Data of outpatient who came to monitor the growth and development were automatically extracted and uploaded from the hospital information system to the database. Furthermore, parents from 31 provinces, autonomous regions or municipalities all over China could upload data to XIGAO database on their mobiles through the QR code displayed in websites, hospitals or during the health education of schools and kindergartens.

In this study, data including sex, date of birth, date of measurement, province, height and weight were collected and analyzed. Cases who were younger than 3 years old or older than 19 years old, lack important information such as sex, height, weight and age, whose height significantly deviated from the growth curve (height SDS without ± 2SD), or who had biologically implausible BMI (BMI z-score without ± 5SD) were removed (6).

This study was approved by the ethic committee of Shijiazhuang Kid Grow Science and Technology Co. Ltd (2021[8]). The information related to patient identification in this study was hidden and was not handed over to the researchers.

### Measurements and Obesity Definition

Height was accurate to 0.1 cm, and weight was accurate to 0.1 kg. BMI was calculated as weight (kg) divided by height squared (m^2^). We calculated the z-score of BMI, height and weight according to Chinese data (7, 8).

For children ≥7 years old, the diagnosis of obesity or overweight was made according to the Chinese standard (9). For children between 3–5 years old, BMI z-score ≥2 was defined as overweight and BMI z-score ≥3 was defined as obesity; while for children between 5–7 years old, BMI z-score ≥1 was defined as overweight and BMI z-score ≥2 was defined as obesity. Underweight children and adolescents were assigned to the normal category when analyzing.

### Statistical Analysis

Age-, sex- and region-standardized prevalence of obesity and overweight were calculated based on 2010 China census population data (10). The trends of prevalence of obesity and overweight and BMI z-score were also calculated separately by sex, age (3–6 years old, 7–11 years old, 12–14 years old and 15–19 years old; children <7 years old were defined as preschool children, children ≥7 years old were defined as school-aged) and region (Central, East, North, Northeast, Northwest, South and Southwest). Since the COVID-19 epidemic in China was brought under control after April 2020 and schools in various regions gradually resumed classes after May 2020 (11); therefore, in order to understand the short-term impact of the epidemic on weight gain in children and adolescents, we compared the data in 2019, the first half of 2020, and the second half of 2020 to evaluate the impact of pandemic. Student’s *t* test and *χ^2^* test were used to compare continuous and categorical variables. Bland-Altman analysis was used to evaluate the consistency of hospital- and mobile-based data. A two-sided *p* value < 0.05 was considered statistically significant. Statistical analyses were conducted using the R software program (version 4.3).

## Results

### Basic Characteristics

**Figure 1:**
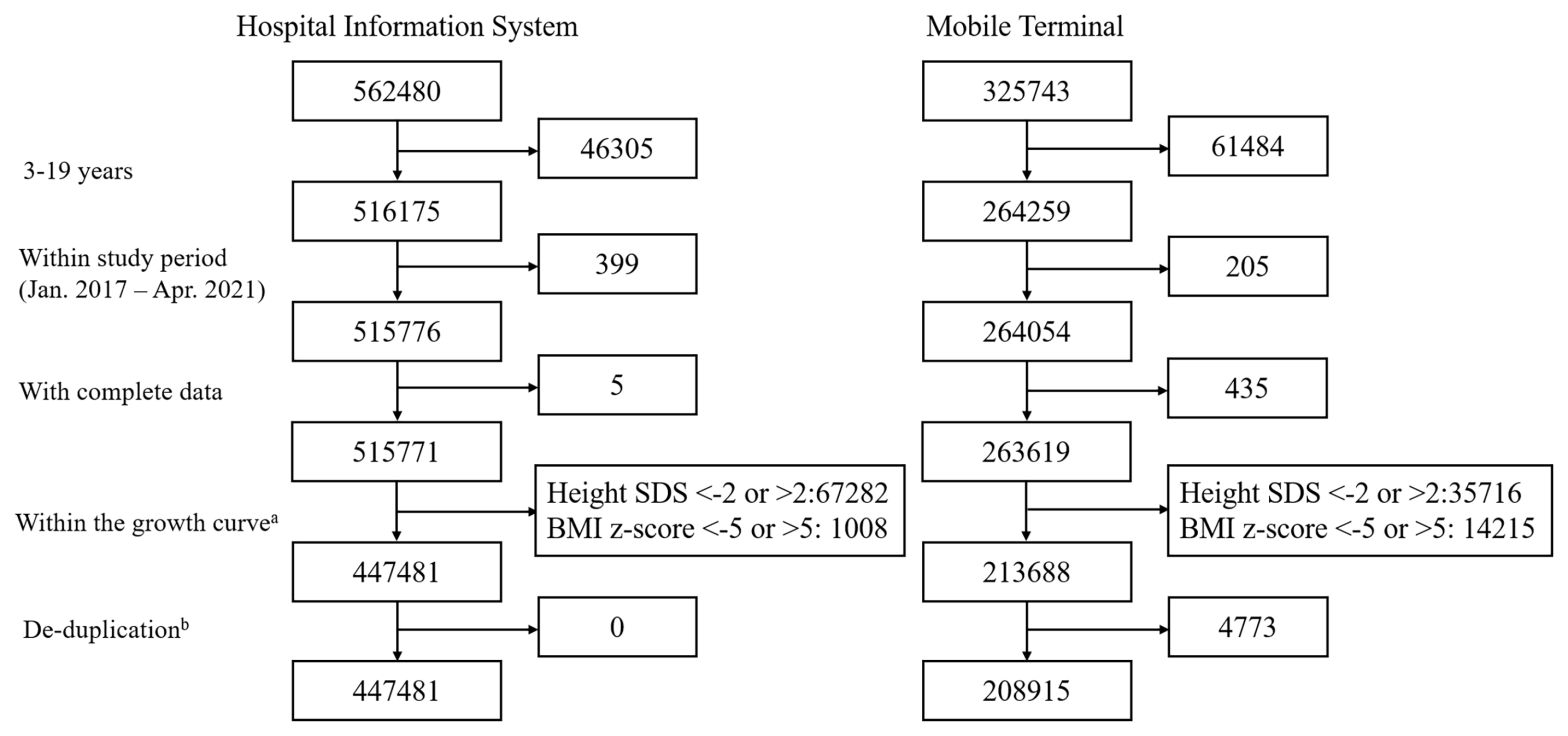
Flow chart of sampling procedure. ^a^To remove the cases that might had growth disorder, we only included the cases whose height age- and sex- specific SDS was between ±2. Then cases with BMI z-score higher than 5 or lower than -5 were excluded in order to excluded incorrect entries and weight abnormalities that might be secondary to other diseases. ^b^Each child had a unique ID. Cases that repeatedly registered data in both the hospital database and the mobile database were regarded as repeated cases, and data from mobiles were excluded.

During the study period, a total of 562840 cases from hospital and 325743 participants from mobiles were enrolled in this study. Finally, a total of 656396 cases were analyzed, including 447481 hospital-measured cases and 208915 parent-reported cases (**Figure 1**), with the mean age of 7.22 ± 3.18 years. **Table 1** showed the general characteristics of the study population.

**Table 1:** General characteristics of the study population.

	Total (n = 656396)	Female (n = 321973)	Male (n = 334423)	*P^#^*
Age (years)	7.22 (3.18)	7.30 (2.96)	7.24 (3.39)	< 0.0001
Age groups (years)				< 0.0001
3–6	363466 (55.4%)	167364 (52.0%)	196102 (58.6%)	
7–11	230412 (35.1%)	133112 (41.3%)	97300 (29.1%)	
12–14	53328 (8.1%)	18353 (5.7%)	34975 (10.5%)	
15–19	9190 (1.4%)	3144 (1.0%)	6046 (1.8%)	
Region^*^				< 0.0001
Central	61046 (9.3%)	29099 (9.0%)	31947 (9.6%)	
East	170941 (26.0%)	83280 (25.9%)	87661 (26.2%)	
North	161040 (24.5%)	77798 (24.2%)	83242 (24.9%)	
Northeast	14202 (2.2%)	6883 (2.1%)	7319 (2.2%)	
Northwest	6809 (1.0%)	3368 (1.0%)	3441 (1.0%)	
South	140042 (21.6%)	73276 (22.8%)	66766 (20.0%)	
Southwest	65651 (10.0%)	32434 (10.1%)	33217 (9.9%)	
BMI z-score	0.18 (1.31)	0.20 (1.30)	0.15 (1.33)	< 0.0001
Obesity status				< 0.0001
Obesity	50534 (7.7%)	22467 (7.0%)	28067 (8.4%)	
Overweight	65750 (10.0%)	30278 (9.4%)	35472 (10.6%)	
Obesity/Overweight	116284 (17.7%)	52745 (16.4%)	53539 (19.0%)	

Data were n (%) or mean (SD). BMI, body-mass index.

^*^36665 cases from mobiles didn’t have exact province. ^#^p value for difference in different sexes.

**Figure 2:**
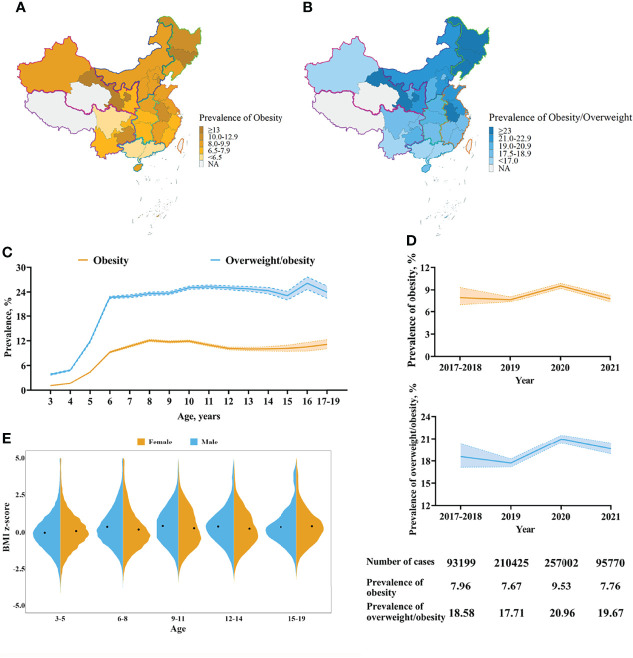
Characteristics of the standardized prevalence of obesity and overweight and BMI z-score. **(A)** The standardized prevalence of obesity by province. **(B)** The standardized prevalence of obesity and overweight by province. The prevalence was standardized by sex and age in different provinces. This study didn’t included children in Taiwan, Hong Kong and Macao. Cases in Tibet Autonomous Region and Qinghai province were limited thus were not included in the map. **(C)** Trajectories of the prevalence of obesity and overweight by age. **(D)** Trends of the prevalence of obesity and overweight. The prevalence was standardized by sex and age. The band indicated 95% CI. **(E)** BMI z-score of different sexes and ages. Black points indicated the mean of BMI z-score of different groups.

The mean BMI z-score of the study population was 0.18 ± 1.31. Girls had higher BMI z-score than that of boys (0.20 ± 1.30 *vs.* 0.15 ± 1.33, *p* < 0.0001). However, the distribution of BMI z-score was different among different age groups between boys and girls (**Figure 2E**). In age group 3-5 years, boys displayed higher BMI z-score than girls (*p* < 0.0001), while BMI z-score of girls was higher than that of boys in age group 6–8 years, 9–11 years, and 12–14 years. Among adolescents aged 15–19, there was no significant statistical difference of BMI z-score between boys and girls.

### Prevalence of Obesity and Overweight

According to Chinese standard, 50534 (7.7%) and 65750 (10.0%) children and adolescents in the study population were obese and overweight, respectively. After adjusting age, sex and region, the standardized prevalence of obesity and obesity/overweight in the study population was 8.05% (95%CI 7.76%–8.39%) and 19.19% (95%CI 18.73%–19.70%).

Boys had higher standardized prevalence of obesity than girls (9.03% [95%CI 8.65%–9.46%] *vs.* 6.91% [95%CI 6.46%–7.47%]). Children in the north area of China presented the highest standardized prevalence of obesity (10.85% [95%CI 9.37%–12.73%]), and followed by the northeast area of China (9.48% [95%CI 8.98%–10.04%]); while the south (6.16% [95% 5.75%–6.61%]) and southwest (6.68% [95%CI 6.41%–7.31%]) region had the lowest obesity prevalence (**Supplementary Table S1** and **Figures 2A, B**).

The obesity prevalence changed with ages. It first increased significantly after the age of four, reached the peak at the age of eight, then slowly declined between 9–14 years old, and finally raised again at the age of 15 (**Figure 2C**). The overall change trend of the prevalence of obesity/overweight was similar to the prevalence of obesity, but it always showed a high level after the age of eight.

### Trends of Obesity Prevalence and BMI z-Score

Despite the significant increase in 2020 due to COVID-19 lockdown, the prevalence of obesity and obesity/overweight among Chinese children and adolescents showed a steadily decrease trend (**Figure 2D**). Compared with 2017–2018, the prevalence of obesity and obesity/overweight in 2019 was decreased by 0.26% and 0.87% respectively. Although increased to 9.53% in 2020, with the increase of 1.86%, the prevalence of obesity returned to 7.76% in the first four months of 2021, which was close to that in 2019.

However, BMI z-score of children and adolescents increased in the past five years (**Supplementary Figure 1**). Compared with the lowest level in 2019, with 0.09 ± 1.35, the mean BMI z-score increased to 0.23 ± 1.34 in 2020 and continued to reach 0.27 ± 1.23 in the first four months of 2021. But the changes of BMI z-score had sex, age and region characteristics, and were related with COVID-19 pandemic shutdown.

For preschool children, a significant increase of the mean of BMI z-score was found in the first four months of 2021; while BMI z-score increased significantly among school-aged children and adolescents in the year of 2020, and a slight decrease was found in 2021 (**Supplementary Table S2**).

**Figure 3:**
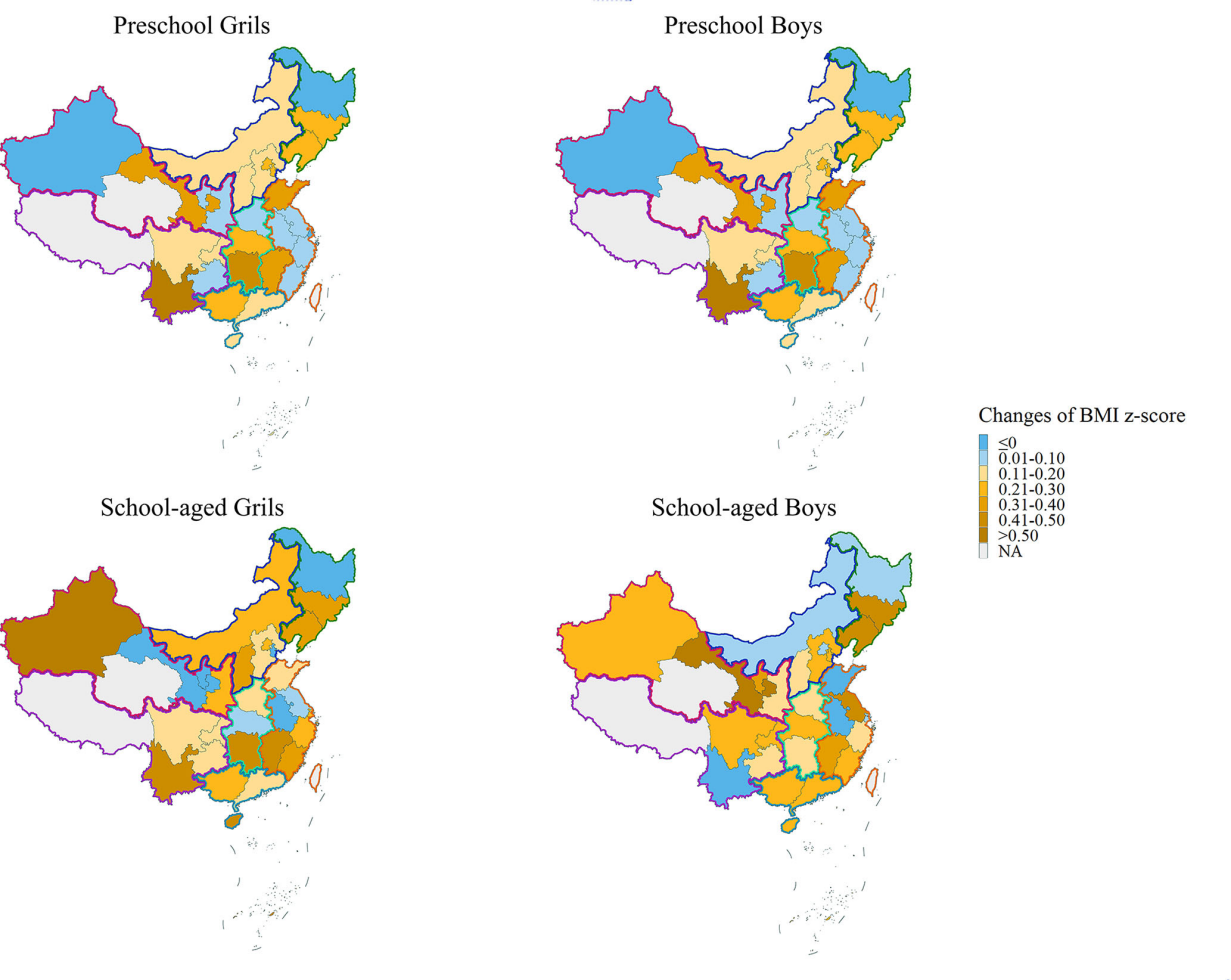
BMI z-score changes during COVID-19 lockdown by province. The changes of the mean BMI z-score in the first half of 2020 and that in 2019.

Further analyzing the influence of COVID-19 lockdown on the changes of BMI z-score, we found that children aged 7–11 years old were most effected by the lockdown with the increase of 0.26 in the mean of BMI z-score in the first half of 2020 compared with 2019, followed by adolescent aged 12–14 years, with the increase of 0.20 (**Table 2**). Preschool children only had relatively slight increase in BMI z-score in the first half of 2020 and returned to the previous level in the second half of 2020. Furthermore, children in the northeast gained the most weight in the first half of 2020, and children in the central, north and southwest regions also experienced an increase of BMI z-score more than 0.20 (**Figure 3**). Notably, the BMI z-score of both preschool and school-aged children in the north area of China increased constantly, even after the year of 2020 (**Supplementary Table S3**).

**Table 2:** BMI z-score changes during COVID-19 shutdown by different ages and regions.

	2019	Jan.-Jun. 2020	Jul. – Dec. 2020	Jan.-Apr. 2021
Age groups (years)				
3–6	0.03 (1.36)	0.12 (1.33)	0.01 (1.29)	0.15 (1.24)
7–11	0.20 (1.34)	0.46 (1.32)	0.39 (1.36)	0.38 (1.23)
12–14	0.19 (1.26)	0.39 (1.35)	0.32 (1.35)	0.32 (1.18)
15–19	0.34 (1.28)	0.45 (1.35)	0.38 (1.27)	0.41 (1.09)
Region				
Central	0.13 (1.31)	0.35 (1.32)	0.24 (1.30)	0.29 (1.16)
East	0.17 (1.30)	0.35 (1.33)	0.31 (1.34)	0.34 (1.19)
North	0.17 (1.34)	0.38 (1.31)	0.21 (1.30)	0.50 (1.22)
Northeast	0.13 (1.40)	0.50 (1.40)	0.30 (1.34)	0.33 (1.29)
Northwest	0.11 (1.23)	0.24 (1.36)	0.33 (1.28)	0.38 (1.26)
South	-0.09 (1.37)	0.08 (1.30)	0.02 (1.33)	0.07 (1.25)
Southwest	-0.02 (1.28)	0.22 (1.29)	0.12 (1.25)	0.14 (1.15)

Data were mean (SD).

### The Accuracy of Parent-Reported Data

To analyze the accuracy of parent-reported data, we compared the duplicate cases between hospitals and mobiles within 30 days. Finally, 4309 cases uploaded data both from hospitals and mobiles within 30 days. When reviewing these data, 3980 (92.3%) cases reported the same BMI through hospitals and mobile terminals, and the differences of BMI of 4096 cases were within 0.20 (95.1%). Furthermore, we found 27 cases (0.6%) whose parent-reported weight value was nearly two times higher than hospital-measured weight value, indicating parents might mistake the unit when uploading the data. There was no significant difference between the BMI z-score of hospital- and mobile-based data according to Bland-Altman analysis (*p* = 0.62) (**Supplementary Figure 2**). Since the proportion of possible errors and bias was low, the parent-reported data from the mobiles was reliable.

### The Characteristics of Parent-reported Measurements in BMI Monitoring

31.8% cases of this study were parent-reported data obtained from the mobiles; therefore, we could evaluate the characteristics of parent-reported data (**Supplementary Table S4**). Parents of preschool children were more inclined to participate in the BMI monitoring study, accounting for 73.9% of the total mobile-based data. Compared with the hospital measurement data, parent-reported data had higher standardized obesity and obesity/overweight prevalence (6.9% [95% CI 6.7%–7.3%] *vs.* 11.8% [95% CI 10.9%–13.0%]; 18.3% [95% CI 17.7%–18.9%] *vs.* 21.7% [95% CI 20.7%–23.0%]). Furthermore, parent-reported data had higher BMI z-score among both preschool and school-aged children (0.07 ± 1.42 *vs.* 0.05 ± 1.21, *p* = 0.0020; 0.43 ± 1.57 *vs.* 0.30 ± 1.25, *p* < 0.0001). However, both mobile-based data and hospital-based data could timely reflect the changes of BMI during pandemic (**Supplementary Table S5**).

## Discussion

This first large-scale study evaluated the changing trend of obesity prevalence and BMI of children and adolescents in different regions of China in the past five years using mobile- and hospital-based data. From 2017 to 2021, the obesity and overweight prevalence of Chinese children and adolescents remained at a stable level and had a slight downward trend; though the lockdown in 2020 led to a significant increase. The COVID-19 epidemic in 2020 had an impact on obesity and weight gain, but there were regional and age differences.

In the past decade, the prevalence of obesity and overweight in children and adolescents in China increased from 15.4% in 2010, to 20.4% in 2014 and 24.7%–25.8% in 2017–2019 (12–14). In our study, the prevalence of obesity and overweight was 19.19%, which was slightly lower compared with that Zhang et al. (12) and 2014 Chinese National Survey on Students’ Constitution and Health (15) found. Since our study included more provinces in the south and southwest area of China and preschool children aged 3–6 years old, their relatively low prevalence of obesity and overweight resulted in a lower overall prevalence than previous studies.

The prevalence of obesity had region-, age- and sex-specific characteristics. Consist with previous studies, Chinese boys and children in northern region of China had higher prevalence of obesity, while children in south and southwest regions of China had lower prevalence (12, 16, 17). Furthermore, even in the same province, there are differences in obesity in different cities, calling for more targeted policies for the controlling of pediatric obesity in China (18). As for the difference between different ages, we found that the obesity prevalence increased significantly after the age of four, while it was relatively low between the ages of 12–15, but then increased again. The increase of obesity prevalence in adolescents older than 16 years old was consistent with the findings among Chinese adults that young adult men had relatively high obesity prevalence (19).

Remarkably, our study found that the prevalence of obesity among Chinese children had entered a stage of stable or even with a slight decline, comparing to the annual growth rate of 0.58% and 0.63% in obesity and overweight respectively in 2010–2014 (17). A decrease of 0.26% in obesity prevalence was found in 2019 compared with 2017–2018. Although experienced a significant increase in 2020, the prevalence of obesity in the first four months of 2021 was 7.79%, which was decreased significantly comparing with the prevalence in 2020, with a decrease of 1.77%. Due to the limited research period and the special impact of the COVID-19 epidemic, we cannot conclude that the obesity problem of Chinese children has been controlled. The study of Yuan et al. (20) among Chinese children and the study of adult population in China both found a slowdown in the rise of obesity prevalence (19), suggesting an optimistic trend of obesity problem among all age groups in China. However, the overall prevalence is still at high point and long-term trend needs to be observed. Furthermore, the huge population of obese and overweight children and adolescents still needs more attention.

Although some studies have focused on the weight gain of children in China during the COVID-19 epidemic (4, 21, 22), there is still a lack of comprehensive national analysis of multiple age groups. To reduce the impact of going out restrictions on the assessment of children’s growth and development, we innovatively use mobiles as a data-source for this nationwide survey. Consist with previous regional studies (4, 5), COVID-19 lockdown led to weight gains among Chinese children, but also had age and region differences.

The age difference in weight gain might be related to the growth characteristics and lifestyles of children of different ages. We found that children and adolescents aged 7–14 years old, mainly children in primary schools and junior middle schools, had the most BMI z-score gain. For school-aged children, the lower the grade, the more time spent in school activities, including physical education classes and extracurricular activities; therefore, the exercise time of primary and junior middle school students was greatly affected by the lockdown. High school students had less activity time at school than younger children in China, so they might be less affected; while preschool children had a lot of non-school activity time, so they were almost unaffected. Findings of other regional studies in China and the United States were consistent with our results (4, 22, 23).

Regional differences in weight gain during COVID-19 lockdown might be affected by various factors such as the severity of the epidemic and differences in local lifestyles. Children in northeast, central, north and southwest area showed a significant increase in BMI z-score in the first half of 2020 and a decline in the second half of the year, which was consistent with the development trend of the epidemic in China. However, the weight of children in the northwest and north increased continuously even after the epidemic, and the mean BMI z-score reached 0.5 in northern China. This suggested that the north and northwest regions are the focus of attention of obesity monitoring in the future.

Conducting family self-assessment was a good way for monitoring weight changes and providing timely interventions based on changes, especially during the epidemic, when the assessment carried out in schools and clinics was restricted. Our study has confirmed the feasibility of using mobiles and the Internet to conduct growth and development assessments for children and adolescents across China. By entering the basic information and anthropometry data, parents can obtain the results of their children’s developmental assessment within a few seconds, avoiding complicated calculations and table lookups that need to be completed by pediatricians. In the future, the online assessment method can be widely promoted as an effective assessment method for children’s growth and development, especially during epidemics and in remote areas. The main concern about the online assessment was the inaccuracy of parent- and self-reported data, which might have bias due to reporting behaviors and systematic measurement differences. Zhou et al. (24) raised concerns about the accuracy of self-reported weight and height of Chinese adolescents, and He et al. (25) found that parent-reported BMI tended to overestimate the prevalence of obesity in children and adolescents. A study of the accuracy of parental measurements of height and weight during COVID-19 found lower BMI at home than in clinics (26). Although the accuracy analysis of this study suggested that the parent-reported data was reliable, we found that the BMI z-score of mobile-based data was higher than those of hospital-based data in this study, especially among school-aged children. Although there was statistical difference among preschool children, the difference was only 0.02. In addition to the possible errors caused by clothing and food intake during the home measurement process, parents of obese children were more inclined to participate in related assessments might be the reason for this difference. Even with bias, a slight overestimation of BMI in obesity assessment helped parents of children at risk of overweight to pay more attention on their diets and lifestyles, and seek help from pediatricians in time. Therefore, parent-reported growth assessment through mobile was a feasible, efficient and timely way with synchronous response, thus it is worthy of being widely promoted. In the future, better health education and measurement teaching will help parents measure their children’s height and weight more accurately.

One of the limitations of this study was that all cases from mobiles were voluntarily participated. Therefore, younger children accounted for a large proportion of the study population. However, when calculating the trend of BMI, children were divided into different age groups, and when calculating the obesity prevalence, we standardized the prevalence according to age, sex and region, so it would not affect the results. In addition, we found that the obesity prevalence of mobile-based data was higher than that of the hospital-based data and other researches (2, 12, 13). The reason for this phenomenon might be that parents of overweight children paid more attention to the assessment of their children’s growth and development and were more inclined to participate in such surveys. Furthermore, this large-scale epidemiological survey didn’t collect information about lifestyles, such as dietary or physical activities, which were important factors affecting BMI. In the future, we will expand the information collected in the database to better investigate possible factors affecting BMI changes.

## Conclusions

In conclusion, the prevalence of obesity and overweight of Chinese children and adolescents showed a slight downward trend though increased in 2020 due to the COVID-19 lockdown. The high obesity prevalence in northern China and the continuous increase of BMI z-score in north and northwest regions called for more region-specific ways for pediatric obesity management. The assessment through mobiles based on parent-reported anthropometry data was a good way for obesity monitoring, especially during epidemics. High BMI z-score increase among primary and junior middle school children suggested more indoor sports education for these children especially during the pandemic.

## Data Availability Statement

The raw data supporting the conclusions of this article will be made available by the authors, without undue reservation.

## Ethics Statement

The studies involving human participants were reviewed and approved by the ethic committee of Shijiazhuang Kid Grow Science and Technology Co. Ltd. Written informed consent from the participants’ legal guardian/next of kin was not required to participate in this study in accordance with the national legislation and the institutional requirements.

## Author Contributions

YY and ZX conceptualized and designed the study, completed the statistical analyses, drafted the initial manuscript, and reviewed and revised the manuscript. MZ and SZ developed the database and supervised the data collection. FL supervised the data collection, contributed to the design of the study, and reviewed and revised the manuscript. ZP and CS contributed to interpretation of the data and extensive revision of the manuscript. JY, JH and TQ assisted with data interpretation and reviewed and revised the manuscript. All authors approved the final manuscript as submitted and agreed to be accountable for all aspects of the work.

## Funding

This study is supported by the National Key Research and Development Program of China, No. 2021YFC2701904, and the Clinical special project of integrated traditional Chinese and Western medicine in 2019, Shanghai Municipal Health Commission, Shanghai Municipal Administrator of Traditional Chinese Medicine. The funder of the study had no role in study design, data collection, data analysis, data interpretation, or writing of the report.

## Conflict of Interest

Authors MZ and SZ were employed by Shijiazhuang Xigao Technology Co. Ltd.

The remaining authors declare that the research was conducted in the absence of any commercial or financial relationships that could be construed as a potential conflict of interest.

## Publisher’s Note

All claims expressed in this article are solely those of the authors and do not necessarily represent those of their affiliated organizations, or those of the publisher, the editors and the reviewers. Any product that may be evaluated in this article, or claim that may be made by its manufacturer, is not guaranteed or endorsed by the publisher.
