# Supplementary material for: Nationwide Trends of Pediatric Obesity and BMI z-Score From 2017-2021 in China: Comparable Findings From Real-World Mobile- and Hospital-Based Data
Source: Front Endocrinol (Lausanne). 2022 May 26;13:859245. doi: 10.3389/fendo.2022.859245 (PMC9204322; doi:10.3389/fendo.2022.859245)
Supplement: Supplementary file 1 [file DataSheet_1.docx]

Supplementary Material

**Supplementary Table S1. Standardized prevalence of obesity and overweight of different regions**

| **Region** | | **Prevalence of obesity*** | **Prevalence of overweight*** |
| --- | --- | --- | --- |
| Central | | 8.17 (7.71–8.68) | 11.04 (10.50–11.63) |
| East | | 8.36 (8.00–8.75) | 11.29 (10.87–11.72) |
| North | 10.85 (9.37–12.73) | | 11.75 (10.10–12.80) |
| Northeast | | 9.48 (8.98–10.04) | 12.27 (11.68–12.90) |
| Northwest | | 8.18 (6.27–10.83) | 12.76 (10.26–15.98) |
| South | | 6.16 (5.75–6.61) | 9.45 (8.92–10.02) |
| Southwest | | 6.68 (6.41–7.31) | 10.79 (10.17–11.46) |

Prevalence was shown as % (95% CI).

* Adjusted by sex and age according to the 2010 China census population data.

**Supplementary Table S2: BMI z-score changes by sex and age**

|  | **Preschool** | | | **School-aged** | | |
| --- | --- | --- | --- | --- | --- | --- |
| Year | Total | Female | Male | Total | Female | Male |
| 2017-2018 | 0.06 (1.20) | 0.02 (1.19) | 0.09 (1.20) | 0.21 (1.29) | 0.29 (1.25) | 0.13 (1.31) |
| 2019 | 0.03 (1.36) | -0.03 (1.34) | 0.08 (1.37) | 0.21 (1.33) | 0.29 (1.31) | 0.13 (1.35) |
| 2020 | 0.06 (1.31) | 0.03 (1.30) | 0.08 (1.31) | 0.41 (1.34) | 0.48 (1.29) | 0.32 (1.40) |
| 2021 | 0.15 (1.24) | 0.12 (1.23) | 0.18 (1.24) | 0.37 (1.22) | 0.46 (1.19) | 0.26 (1.24) |

Data were mean (SD).

**Supplementary Table S3: BMI z-score changes by regions**

|  | **Preschool** | | | | | | | **School-aged** | | | | | | |
| --- | --- | --- | --- | --- | --- | --- | --- | --- | --- | --- | --- | --- | --- | --- |
|  | Central | East | North | Northeast | Northwest | South | Southwest | Central | East | North | Northeast | Northwest | South | Southwest |
| 2017-2018 | 0.04 (1.30) | -0.01 (1.21) | 0.18 (1.16) | 0.16 (1.27) | 0.06 (1.24) | -0.11 (1.27) | -0.03 (1.13) | 0.26 (1.37) | 0.23 (1.26) | 0.28 (1.31) | 0.31 (1.36) | 0.07 (1.27) | 0.05 (1.31) | 0.24 (1.2) |
| 2019 | 0.04 (1.30) | 0.10 (1.34) | 0.13 (1.32) | 0.09 (1.39) | -0.02 (1.19) | -0.13 (1.39) | -0.15 (1.29) | 0.21 (1.30) | 0.23 (1.26) | 0.33 (1.39) | 0.27 (1.40) | 0.21 (1.25) | 0.01 (1.30) | 0.18 (1.25) |
| 2020 | 0.14 (1.30) | 0.12 (1.33) | 0.13 (1.26) | 0.11 (1.25) | 0.06 (1.29) | -0.09 (1.32) | 0.00 (1.23) | 0.38 (1.30) | 0.44 (1.32) | 0.55 (1.36) | 0.56 (1.41) | 0.49 (1.30) | 0.23 (1.29) | 0.38 (1.28) |
| 2021 | 0.13 (1.14) | 0.27 (1.23) | 0.43 (1.20) | 0.16 (1.28) | 0.32 (1.24) | -0.08 (1.28) | 0.00 (1.13) | 0.37 (1.17) | 0.38 (1.17) | 0.57 (1.25) | 0.40 (1.29) | 0.47 (1.30) | 0.19 (1.22) | 0.32 (1.16) |

Data were mean (SD).

**Supplementary Table S4. General characteristics different data resource**

|  | **Hospital**  **(n = 447481)** | **Mobile terminal**  **(n = 208915)** | ***P^#^*** |
| --- | --- | --- | --- |
| Female | 228585 (51.1%) | 93388 (44.7%) | < 0.0001 |
| Age (years) | 7.67 (3.20) | 6.26 (2.91) | < 0.0001 |
| Age groups (years) |  |  | < 0.0001 |
| 3–6 | 209033 (46.7%) | 154433 (73.9%) |  |
| 7–11 | 189736 (42.4%) | 40676 (19.5%) |  |
| 12–14 | 42390 (9.5%) | 10938 (5.2%) |  |
| 15–19 | 6322 (1.4%) | 2868 (1.3%) |  |
| Region^*^ |  |  | < 0.0001 |
| Central | 47121 (10.5%) | 13925 (6.7%) |  |
| East | 130928 (29.3%) | 40013 (19.2%) |  |
| North | 125233 (28.0%) | 35807 (17.1%) |  |
| Northeast | 6449 (1.4%) | 7753 (3.7%) |  |
| Northwest | 5288 (1.2%) | 1521 (0.7%) |  |
| South | 75733 (16.9%) | 64309 (30.8%) |  |
| Southwest | 56729 (12.7%) | 8922 (4.3%) |  |
| BMI z-score | 0.18 (1.24) | 0.16 (1.47) | < 0.0001 |
| Obesity status |  |  | < 0.0001 |
| Obesity | 33578 (7.5%) | 16956 (8.1%) |  |
| Overweight | 48025 (10.7%) | 17725 (8.5%) |  |
| Obesity/Overweight | 81603 (18.2%) | 34681 (16.6%) |  |

Data were n (%) or mean (SD). BMI=body-mass index.

^*^ 36665 cases from mobile terminal didn’t have exact province. ^#^ *p* value for difference in different sexes.

**Supplementary Table S5: BMI z-score changes during COVID-19 lockdown by different data resources**

|  | **Total** | | **Hospital** | | **Mobile Terminal** | |
| --- | --- | --- | --- | --- | --- | --- |
| Year | Preschool | School-aged | Preschool | School-aged | Preschool | School-aged |
| 2017–2018 | 0.06 (1.20) | 0.21 (1.29) | 0.06 (1.16) | 0.19 (1.23) | 0.06 (1.33) | 0.34 (1.52) |
| 2019 | 0.03 (1.36) | 0.21 (1.33) | -0.02 (1.21) | 0.15 (1.23) | 0.06 (1.45) | 0.41 (1.64) |
| Jan. – Jun. 2020 | 0.12 (1.33) | 0.45 (1.32) | 0.14 (1.24) | 0.42 (1.23) | 0.12 (1.39) | 0.51 (1.54) |
| Jul. – Dec. 2020 | 0.01 (1.29) | 0.38 (1.36) | 0.03 (1.25) | 0.37 (1.30) | -0.06 (1.38) | 0.42 (1.60) |
| Jan. – Apr. 2021 | 0.15 (1.24) | 0.37 (1.22) | 0.15 (1.18) | 0.36 (1.18) | 0.16 (1.36) | 0.41 (1.46) |

Data were mean (SD).


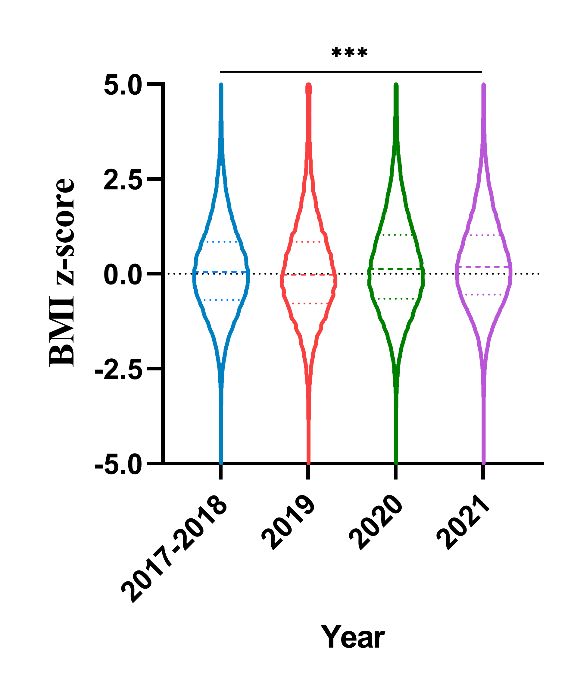


**Supplementary Figure S1: BMI z-score changes from 2017 to 2021.**

*** *p* < 0.001


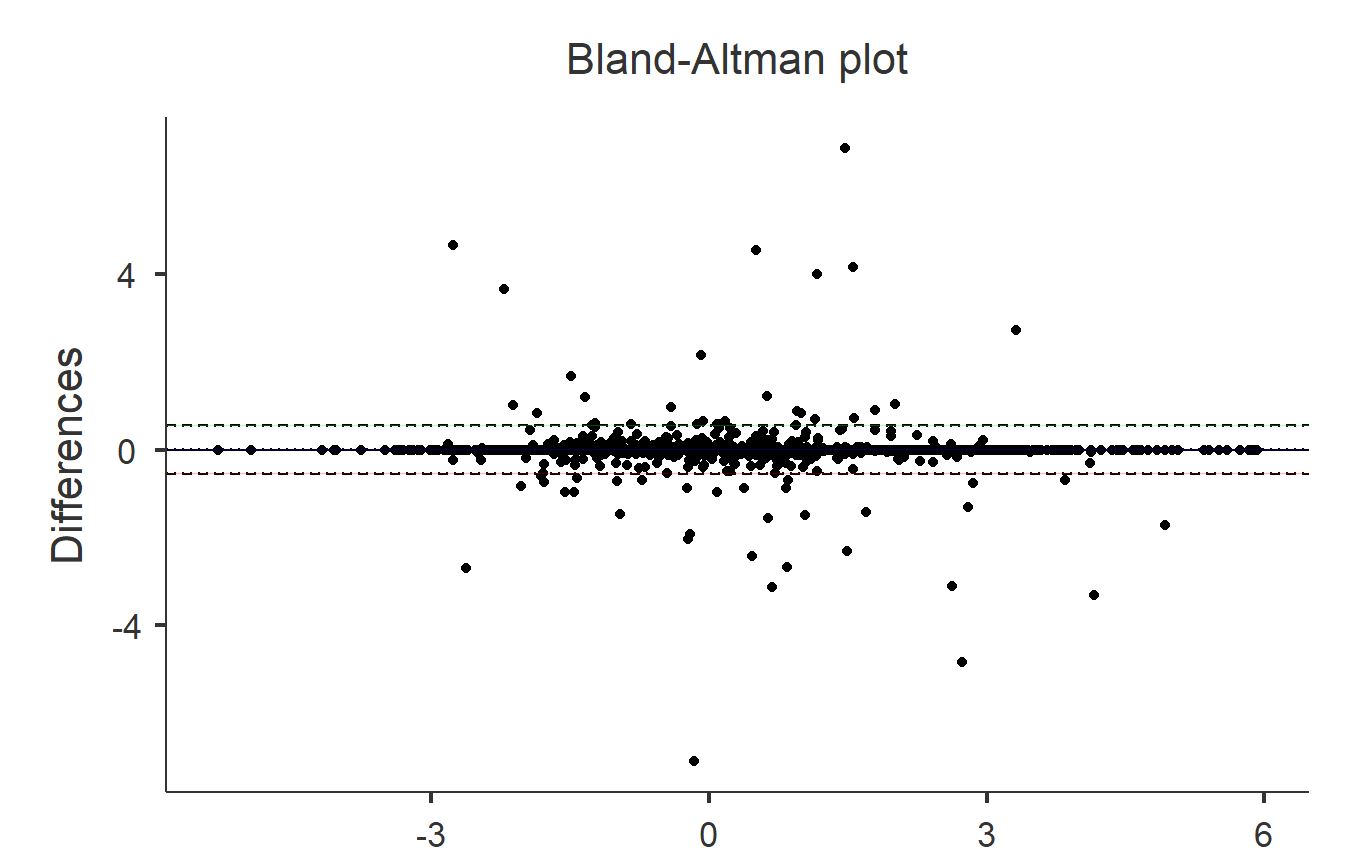


**Supplementary Figure S2: Bland-Altman analysis of duplicated data**
